# Clinical Variables Associated With Grade III and IV Intraventricular Hemorrhage (IVH) in Preterm Infants Weighing Less Than 750 Grams

**DOI:** 10.7759/cureus.40471

**Published:** 2023-06-15

**Authors:** Kiran S Depala, Soumini Chintala, Swosti Joshi, Shaaista Budhani, Nihal Paidipelly, Bansari Patel, Alok Rastogi, Nimisha Madas, Revanth Vejju, Janardhan Mydam

**Affiliations:** 1 Department of Public Health and Social Justice, Saint Louis University, St. Louis, USA; 2 Department of Pediatrics, Phoenix Children's Hospital, Phoenix, USA; 3 Department of Neonatology, John H. Stroger, Jr. Hospital of Cook County, Chicago, USA; 4 Department of Chemistry, Case Western Reserve University, Cleveland, USA; 5 School of Medicine, American University of Barbados, Bridgetown, BRB; 6 Department of Internal Medicine, Northwestern Medicine McHenry Hospital, McHenry, USA; 7 Department of Biology, New Jersey Institute of Technology, Newark, USA

**Keywords:** birth weight below 750 grams, neonates, retrospective study, gestational age, birth weight, hematocrit, intraventricular hemorrhage (ivh), extremely preterm infants

## Abstract

Background: Despite innovative advances in neonatal medicine, intraventricular hemorrhage (IVH) continues to be a significant complication in neonatal intensive care units globally.

Objective: The study aimed to discern the variables heightening the risk of severe IVH (Grade III and IV) in extremely premature infants weighing less than 750 grams. We postulated that a descending hematocrit (Hct) trend during the first week of life could serve as a predictive marker for the development of severe IVH in this vulnerable population.

Methods: This retrospective case-control study encompassed infants weighing less than 750 grams at birth, diagnosed with Grade III and/or IV IVH, and born in a tertiary center from 2009 to 2014. A group of 17 infants with severe IVH was compared with 14 gestational age-matched controls. Acid-base status, glucose, fluid goal, urine output, and nutrient (caloric and protein) intake during the first four days of life were meticulously evaluated. Statistically significant variables from baseline data were further analyzed via univariable and multivariable logistic regression analyses, ensuring control for potential confounding variables.

Results: The univariate logistic regression model delineated odds ratios (ORs) of 0.842 for day 2 average Hct (confidence interval [CI], 0.718-0.987) and 0.16 for urine output on day 3 (CI, 0.024-1.056), with the remaining six variables demonstrating no significant association. In the post-multivariable regression analysis, day 2 Hct was the only significant variable (OR, 0.731; 95% CI, 0.537-0.995; P=0.04). The receiver operating characteristic (ROC) curve analysis portrayed an area under the curve of 71% for the day 2 Hct variable.

Conclusion: The study revealed that a dip in Hct on day 2 of life augments the likelihood of Grade III and IV IVH among extremely premature infants with a birth weight of less than 750 grams. This insight amplifies our understanding of risk factors associated with severe IVH development in extremely preterm infants, potentially aiding in refining preventive strategies and optimizing clinical management and treatment of these affected infants.

## Introduction

Intraventricular hemorrhage (IVH), a prevalent complication linked with preterm birth, is of substantial concern due to its potential to engender profound neurodevelopmental deficits and permanent disabilities, such as deafness and blindness, in preterm infants [1-5]. It is estimated that approximately 20-25% of all preterm infants born before 28 weeks of gestational age suffer from IVH [2,6]. The occurrence of IVH is notably higher in infants with decreased birth weight or gestational age [7]. The late 20th century observed marked reductions in IVH incidence, owing to enhancements in obstetric, prenatal, and neonatal care [3,5,8]. However, parallel advancements have also improved survival rates in extremely low birth weight (ELBW) (<800 g) and very low gestational age (<26 weeks) infants, inadvertently escalating the risk of severe IVH in newborn infants [6,9,10].

IVH typically originates in the periventricular germinal matrix of preterm infants, a region rich in glial and neuronal precursor cells in the developing brain, within the first 48 hours of life [1,6]. The pathogenesis of IVH is multifactorial and complex, with numerous environmental factors possibly contributing to its development [1,6]. Specific predisposing factors include the innate fragility of germinal matrix vasculature, fluctuations in cerebral blood flow (CBF), and anomalies in platelet function and coagulation [1]. It has been observed that the severity of IVH is directly proportional to the risk of associated complications and long-term neonatal outcomes, with as many as 90% of infants with Grade IV IVH at risk [11]. The influence of shared biological pathways and risk factors on severe IVH is yet to be definitively excluded. Certain interventions like antenatal steroid therapy and cesarean birth have demonstrated efficacy in mitigating the risk of severe IVH [12-14]. Conversely, conditions like hypotension and hypercapnia could augment the risk of IVH by influencing CBF [15-17]. Similarly, the practice of delayed cord clamping which can increase blood volume is postulated to diminish IVH incidence [18]. Despite these findings, our understanding of the risk factors underpinning IVH and its clinical management remains limited.

Hematocrit (Hct), defined as the fraction of blood volume constituted by red blood cells, is often considered a surrogate marker for blood volume. Concordantly, several studies have probed into the direct relationship between Hct and IVH incidence [17,19-21]. A substantial recent study including ELBW infants (92 with IVH, 153 without IVH) highlighted that lower Hct (<45%) in the initial days of life (DOL) could potentially amplify the risk of IVH more than twofold (OR = 2.38, 95% CI = 1.19-4.76) [20]. The study further identified both C-section and gestational age as independent predictors of IVH. However, investigations into the impact of Hct in the initial DOL on the incidence of severe IVH (Grade III/IV) remain scant. The present study seeks to identify clinical variables that heighten the risk of severe IVH in preterm neonates. Our hypothesis posits that a decreased Hct during the first week of life augments the risk of severe IVH (Grade III/IV) in extremely premature infants (<750 g).

## Materials and methods

Study design and participants

**Figure 1:**
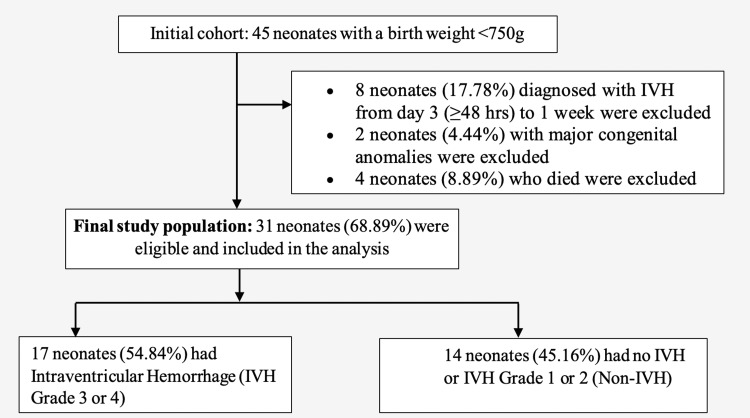
Flowchart of study population selection

This investigation adopted a case-control study design, targeting very low birth weight (VLBW) infants diagnosed with Grade III and/or Grade IV IVH between 2009 and 2014 at a tertiary center. The study's subjects were juxtaposed against gestational age-matched control infants. Inclusion criteria for the infants were a birth weight of less than 750 grams and a diagnosis of Grade III and/or Grade IV IVH within the first week of life (Figure 1). Diagnosis and grading of IVH were performed by a certified pediatric radiologist through cranial sonography. The Papile classification was employed for grading IVH, where Grade III refers to more than 50% occupancy of lateral ventricle volume coupled with ventricular dilatation, and Grade IV denotes the existence of infarction and/or hemorrhage in the periventricular white matter [22].

Data collection

Baseline maternal data including age, ethnicity, prenatal care, antenatal steroid, antibiotic and magnesium sulfate use, mode of delivery, premature rupture of membranes (PROM), and parity were collated. Neonatal data comprising gestational age, gender, birth weight, admission temperature, and Apgar scores at one and five minutes post-delivery were also collected. Rigorous monitoring of the newborn infants facilitated the documentation of data pertaining to acid-base status, glucose levels, fluid goal, urine output, calorie intake, and protein intake during the first four days of hospitalization. The focus on data from the first four DOL stems from the average day of diagnosis for Grade III IVH in our sample being 3.6 DOL. We included infants who were diagnosed with IVH within the first 48 hours of life (equivalent to DOL 3 to DOL 4). Thus, this timeframe allowed us to evaluate how variable differences within this period may influence or predict the grade of IVH (the range of days when head ultrasound was performed extended from day 1 to day 9).

Inclusion and exclusion criteria

The inclusion criteria for the study were categorized into three distinct domains. Primarily, infants with a birth weight of up to 750 grams were considered eligible. Secondly, the timing of diagnosis was crucial; only those diagnosed with IVH within the first 48 hours of life were included. Finally, the study encompassed infants who survived beyond the immediate newborn period.

Conversely, certain conditions warranted participant exclusion. Infants diagnosed with IVH from day 3, or more than 48 hours after birth, through to the first week of life were excluded. Additionally, infants exceeding a birth weight of 750 grams were deemed ineligible. Neonates identified with any congenital anomalies were excluded from the study, and any infants not surviving the immediate newborn period were also omitted from the research.

Statistical analysis

The analytical plan comprised a comprehensive comparative assessment of clinical variables among case and control cohorts. Categorical data were delineated as proportions, while the distribution of continuous variables was appraised using the Shapiro-Wilk test. We presented parametric data as means and standard deviations (SDs), whereas non-parametric data were expressed as medians and interquartile ranges (IQRs).

The chi-square test or Fisher's exact test was utilized for the analysis of categorical variables, while the Student's t-test was applied for parametric variables. Non-parametric variables were examined using the Mann-Whitney U test or the Kruskal-Wallis test. Confidence intervals were set at a 95% significance level, and a p-value threshold of 0.05 was adopted to establish statistical significance.

For binary outcome variables, we computed odds ratios (ORs) to express effect estimates. Variables displaying significant correlation with severe IVH in the univariable analysis were advanced to the multivariable analysis phase. A binary logistic regression model was employed to assess the association between the binary outcome variable and the primary independent variable while adjusting for the effects of potential confounders.

Given that the diagnosis of IVH was ascertained on day 3, our logistic regression model included only those clinical variables from day 2 that demonstrated statistical significance. This approach facilitated the evaluation of the predictive capabilities of the independent variables based on measurements acquired prior to IVH diagnosis.

Subsequently, the Mantel-Haenszel (MH) method was implemented to detect variations in ORs across distinct subgroups of potential confounders. In the event of detecting effect modification, strata-specific ORs were additionally reported. All statistical analyses were carried out using the SAS 9.4 software (SAS Institute, Inc., Cary, North Carolina).

## Results

Participant profile and baseline characteristics

Throughout the five-year study duration, 17 infants diagnosed with severe IVH and 14 gestational age-matched control infants met the inclusion criteria. Upon evaluating the baseline demographics and clinical characteristics of both mothers and their infants, no statistically significant disparities emerged between the IVH and non-IVH infant groups (Table 1). However, several trends were discernible.

**Table 1:** Baseline demographics and clinical characteristics of mother and infant population *Birth weight <750 g. †Data were not available for all patients. ANS: antenatal steroids; BW: birth weight; C-section: cesarean section; DOL: days of life; IVH: intraventricular hemorrhage; Mg: magnesium; GA: gestational age; PNC: postnatal care; PROM: prolonged rupture of membranes

Characteristics	IVH (Grade III or IV); N=17	Non-IVH; N=14	p-value
Maternal age (years), mean (SD)	25.12 (5.42)	28.71 (7.79)	0.140
Ethnicity†, n (%)			
- White	0 (0.00)	2 (14.29)	0.500
- Black	10 (76.92)	8 (57.14)	-
- Hispanic	3 (23.08)	4 (28.57)	-
Sex of infant, n (%)			
- Female	7 (41.18)	9 (64.29)	0.200
PNC†, n (%)			
- Yes	12 (80.0)	11 (78.57)	1.000
- No	3 (20.0)	3 (21.43)	-
ANS, n (%)			
- Yes	10 (58.82)	12 (85.71)	0.132
- No	7 (41.18)	2 (14.29)	-
Number of doses, n (%)			
- 0	7 (41.18)	2 (16.67)	0.288
- 1	5 (29.41)	3 (25.00)	-
- 2	5 (29.41)	7 (58.33 )	-
Chorioamnionitis, n (%)			
- Yes	3 (17.65)	2 (14.29)	1.000
- No	14 (82.35)	12 (85.71)	-
Antibiotic treatment, n (%)			
- Yes	10 (66.67)	8 (57.14)	0.597
- No	5 (33.33)	6 (42.86)	-
Mg treatment, n (%)			
- Yes	4 (28.57)	7 (53.85)	0.182
- No	10 (71.43)	6 (46.15)	-
Outborn, n (%)			
- Yes	10 (58.82)	3 (21.43)	0.036
- No	7 (41.18)	11 (78.57)	-
PROM duration (hours), median (range)	0 (0-240)	0 (0 – 360)	0.574
C-section, n (%)			
- Yes	9 (52.94)	10 (71.43)	0.293
- No	8 (47.06)	4 (28.57)	-
Multiple births, n (%)			
- Yes	6 (35.29)	2 (14.29)	0.240
- No	11 (64.71)	12 (85.71)	-
BW (g), mean (SD)	588.9 (94.99)	610.40 (76.83)	0.500
GA (weeks), mean (SD)	24.06 (1.44)	24.36 (0.93)	0.720
Apgar 1-minute, n (%)			
- Poor	12 (80.0)	10 (71.43)	0.680
- Good	3 (20.0)	4 (28.57)	-
Apgar 5-minute, n (%)			
- Poor	8 (53.33)	6 (42.86)	0.570
- Good	7 (46.67)	8 (57.14)	-
Admission temperature (°C), median (range)	36.60 (33.00-37.20)	36.60 (35.80-37.00)	0.790
Surfactant use			
- Yes	60 (99.77)	29 (100.00)	1.00
- No	2 (3.23)	0 (0.00)	-
DOL at the start of treatment, mean (SD)	4 (1-52)	5 (2-44)	0.40

Mothers delivering IVH infants exhibited a slightly lower mean age compared to those giving birth to non-IVH infants (25.1 vs 28.7 years) and were predominantly of Black ethnicity (76.9% vs 57.1%). It is noteworthy that the vast majority of mothers across both cohorts had received postnatal care (PNC). Regarding therapeutic measures, a substantial proportion of mothers giving birth to IVH infants had been administered magnesium and antenatal steroids. Intriguingly, a higher fraction of non-IVH infants were delivered via cesarean section.

In terms of neonatal characteristics, female infants constituted a majority within the non-IVH group (64.3% vs 41.2%). Infants diagnosed with IVH were slightly underweight and exhibited a marginally reduced average gestational age compared to non-IVH infants. Additionally, IVH infants demonstrated compromised Apgar scores at one and five minutes post-delivery.

Follow-up characteristics of infants

The univariate analysis of follow-up characteristics unveiled significant associations between distinct variables at specific stages (Table 2). On day 1, IVH infants demonstrated a significantly elevated mean PCO2 (partial pressure of carbon dioxide) level (45.15±12.00 vs. 37.93±5.06, P=0.03). On the second day, a higher calorie intake was observed among IVH infants. Notably, Hct levels on day 2 were significantly lower in IVH infants compared to non-IVH infants (35.42±5.08 vs. 40.82±6.74, P=0.02). This was succeeded by a notable decline in urine output among IVH infants on day 3 (3.45±1.18 vs. 4.83±0.83, P=0.01). On the final day of the observational period, the distribution of average base excess revealed a marked difference between the two groups (-11.31±3.47 vs. -6.44±4.00, P=0.02).

**Table 2:** Follow-up clinical characteristics of the infant population IVH: intraventricular hemorrhage; PCO2: partial pressure of carbon dioxide in blood; SD: standard deviation; Hct: hematocrit

Clinical variable	IVH (Grade III or IV); N =17	No IVH; N = 14	p-value
Day 1			
PCO2, mean (SD)	45.15 (12.00)	37.93 (5.06)	0.03
Hct, mean (SD)	37.26 (7.87)	40.03 (9.25)	0.375
Calorie intake, mean (SD)	2.86 (11.28)	28.19 (6.71)	0.222
Protein intake, median (range)	2.20 (1.40-3.20)	2.40 (1.70-4.40)	0.281
Serum glucose, mean (SD)	115.57 (44.16)	92.19 (33.83)	0.115
Base excess, median (range)	-7.00 (-19.33--2.00)	-7.45 (-10.00--2.00)	1.000
Urine output, mean (SD)	3.73 (1.77)	2.98 (1.50)	0.292
Day 2			
PCO2, mean (SD)	41.65 (10.07)	39.97 (12.26)	0.678
Hct, mean (SD)	35.42 (5.08)	40.82 (6.74)	0.02
Calorie intake, mean (SD)	44.32 (17.47)	32.23 (7.20)	0.03
Protein intake, mean (SD)	2.25 (0.68)	2.51 (0.62)	0.323
Serum glucose, median (Range)	123.50 (78.00-315.75)	119.90 (44.33-193.00)	0.311
Base excess, median (Range)	-7.00 (-16.00--4.00)	-7.45 (-10.80--5.00)	0.781
Urine output, mean (SD)	3.76 (1.64)	3.47 (0.95)	0.609
Day 3			
PCO2, mean (SD)	57.11 (17.56)	55.28 (9.91)	0.756
Hct, median (Range)	37.05 (28.33-602.83)	38.40 (30.80-52.00)	0.286
Calorie intake, mean (SD)	55.31 (14.89)	40.95 (17.28)	0.09
Protein intake, mean (SD)	2.09 (1.21)	3.21 (1.36)	0.08
Serum glucose, median (Range)	146.25 (81.63-195.33)	108.20 (48.00-288.75)	0.237
Base excess, mean (SD)	-9.54 (4.67)	-7.74 (2.22)	0.248
Urine output, mean (SD)	3.45 (1.18)	4.83 (0.83)	0.01
Day 4			
PCO2, mean (SD)	58.95 (5.46)	41.49 (27.25)	0.094
Hct, median (range)	34.50 (28.00-38.67)	40.25 (0.00-50.50)	0.340
Calorie intake, mean (SD)	45.58 (15.65)	39.90 (23.04)	0.670
Protein intake, mean (SD)	2.11 (1.04)	2.98 (1.76)	0.392
Serum glucose, mean (SD)	147.20 (54.92)	90.46 (56.56)	0.06
Base excess, mean (SD)	-11.31 (3.47)	-6.44 (4.00)	0.02
Urine output, mean (SD)	3.88 (1.39)	3.38 (1.91)	0.592

Multivariable predictors of severe IVH

**Figure 2:**
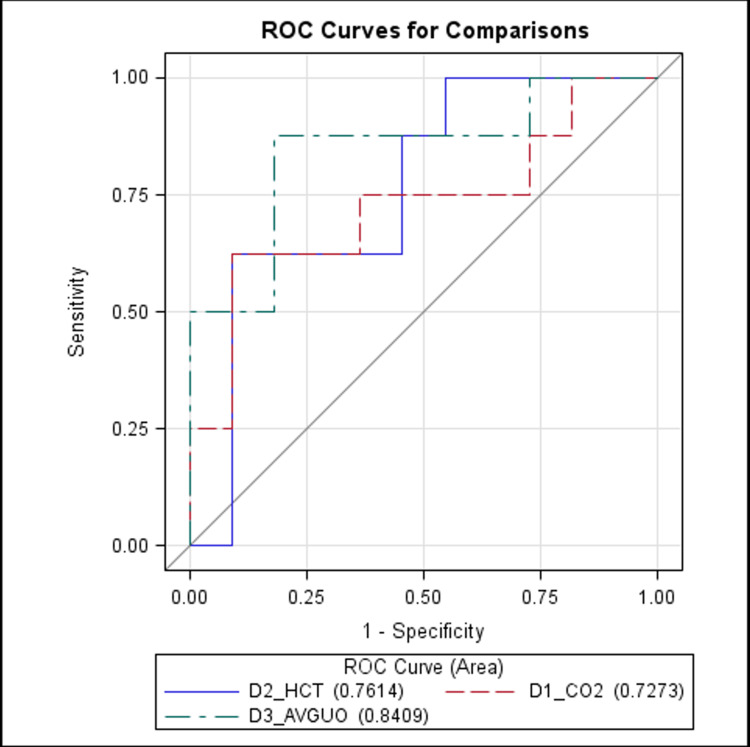
ROC curve analysis for day 2 average Hct, day 1 average PCO2, and day 3 urine output D1: day 1; D2: day 2; D3: day 3; Hct: hematocrit; PCO2: partial pressure of carbon dioxide; ROC: receiver operating characteristic; UO: urine output

Only significant baseline and follow-up variables were considered for the multivariable binary regression association analysis, employed for predicting severe IVH (Table 3). Following the multivariate regression analysis, day 2 average Hct emerged as the only significant variable (Table 3). Specifically, for every one-unit increase in Hct level on day 2, the risk of severe IVH decreased by 27% (OR=0.731, 95% CI = 0.537-0.995). The receiver operating characteristic (ROC) curve analysis of day 2 average Hct levels further corroborated these findings, presenting an area under the curve of 76.4% (Figure 2).

**Table 3:** Prediction of IVH using multivariable logistic regression analysis OR: odd's ratio; CI: confidence interval; Ref: benchmark condition for comparison; Hct: hematocrit Models lacking an OR value are marked with a dash ("-")

Clinical variable	Model 1 OR (95% CI)	Model 1 p-value	Model 2 OR (95% CI)	Model 2 p-value	Model 3 OR (95% CI)	Model 3 p-value	Model 4 OR (95% CI)	Model 4 p-value
JSH/outborn (Ref: Yes)	1.007 (0.127-7.989)	0.9950	0.695 (0.069-7.027)	0.7581	1.101 (0.124-9.761)	0.9313	0.911 (0.067-12.378)	0.9442
Day 2 Hct	0.781 (0.607-1.005)	0.0548	0.782 (0.607-1.006)	0.0558	0.731 (0.542-0.986)	0.0401	0.704 (0.514-0.963)	0.0283
Day 2 calorie intake	1.100 (0.990-1.223)	0.0748	1.098 (0.982-1.226)	0.0996	1.098 (0.986-1.223)	0.0892	1.091 (0.962-1.236)	0.1737
Birth weight (Ref: ≥550 g)	-	-	5.732 (0.535-61.420)	0.1491	-	-	9.540 (0.686-132.776)	0.0932
Gestational age (Ref: ≥25 weeks)	-	-	-	-	0.243 (0.010-5.676)	0.3792	0.092 (0.002-3.812)	0.2092

## Discussion

Principal findings

Our retrospective study explores the relationship between Hct and the occurrence of severe IVH (Grade III and Grade IV). This was done with the purpose of filling the current shortage of information on this clinical variable as a predictor of IVH. Current literature points to an abundance of information detailing the connection between clinical indicators PCO2 and calorie intake as predictors of IVH. Hct, however, is little explored and found through our work to be a more directly connected indicator of potential IVH risk.

The present understanding of the role of PCO2 as a predictor stems from findings regarding elevated PCO2 levels being significantly associated with IVH group preterm and low/ELBW infants in comparison to the control groups [23-25]. Vasodilation as a result of this hypercapnia leads to an increase in CBF that predisposes fragile blood vessels in the germinal matrix to rupturing [26]. In premature infants, autoregulatory mechanisms are underdeveloped and cannot compensate for these fluctuations in CBF-increasing the risk of IVH [27]. Further studies concluded that a PCO2>45 mmHg is also associated with loss of cerebral autoregulation and with the potential to induce IVH [28] while hypercarbia is associated with stronger PCO2 cerebral vasoreactivity in preterm infants [29]. Moving forward, a randomized trial conducted on ELBW infants concluded that poor neurodevelopmental outcomes related to elevated PCO2 are due to IVH (p<0.01) and birthweight (p<0.001) [25]. In our study, day 2 PCO2 was found to be significantly different among study and control populations from hypothesis testing. However, we did not find any association after univariate and multivariate analysis. Despite this, it trended toward significance which is consistent with previous work.

Results in the context of what is known

Multiple scientific studies have shown the importance of caloric intake within proper neurodevelopment. Caloric deprivation has been found to be a significant risk factor [30] as work has shown that infants with slower head growth often have lower caloric intake levels [31]. Continually, a positive association has been highlighted between nutrition and brain volume with nutritional intake having the largest statistically significant impact on brain volume [32]. In our study, cases and control groups had caloric intake within normal ranges, but controls had higher caloric intake levels compared to case group infants. Univariate and multivariate analysis did not show any significant association between IVH and caloric intake, unlike literature data.

With regards to Hct, our study shows after multivariate analysis that a day 2 Hct level of 35.42 is useful as a predictor for IVH in ELBW babies. In previous literature, low Hct is demonstrated to be a useful predictor for moderate-severe grade IVH [2,33,34]. The majority of these studies, however, were conducted on <1500 g and <1000 g babies while very little work was conducted on <750 g babies. In contrast to our study, none of this work discussed the accuracy (sensitivity and specificity) of using Hct as a test to predict IVH much before head ultrasound can predict it to point toward the potential earlier implementation of neuroprotective measures. As a result, our work looks to highlight the benefits of using Hct with regards to its application in screening for IVH in ELBW infants (<750 g) and toward earlier screening of IVH for preterm infants in general before day 3 as per current protocol.

Advantages of Hct

Hct provides several advantages as a diagnostic tool for IVH in comparison to the current gold standard-cranial ultrasounds. In ELBW babies, IVH occurs much earlier in comparison to VLBW babies. In a study comparing IVH timing between 500-700 g birth weight babies and 700-1500 g birth weight babies, 62% of cases occurred in the first 18 hours of life in the 500-700 gram birth weight babies group in comparison to 13% in the >700 g birth weight babies group [35]. Although the protocol of performing a head ultrasound on day 3 and day 7 can pick up the maximum number of IVH cases in >700 g birth weight babies, it can delay the diagnosis of IVH in <700 g birth weight babies.

From the standpoint of VLBW infants, it has been found that in <1500 gram birth weight babies, approximately 48% of IVH cases occurred in the first six hours after birth. This occurrence of early-stage IVH has pointed to this initial six-hour window as being valuable in diagnosing and preventing further progression of IVH [36], pointing to the use of Hct as a screening tool in this window much earlier than the current protocol of a day 3 cranial ultrasound. Further work has shown that in VLBW infants, low-grade (I and II) IVH is not detected with high sensitivity via cranial ultrasound. This bolsters the argument for the use of Hct in another scenario as a possible diagnostic tool for early-stage IVH [37].

Backed up by further literature showing that the cranial ultrasound has zero sensitivity in picking up Grade II IVH (no ventricular dilation) [38], progression toward Grade III and/or IV IVH is a concern in both ELBW and VLBW babies if proper neuroprotective measures are not taken. We found that a day 2 Hct of 35.42 has an 82.35% sensitivity and 14.29% specificity to detect IVH. Therefore, employing Hct as a predictor will not only enable us to detect IVH earlier but also to implement neuroprotective measures that prevent the further progression of IVH. This will aid in improving long-term neurodevelopmental outcomes. Some neuroprotective measures that can be implemented are a midline head position with head elevation alongside a low noise environment [23,39].

Comparison with earlier studies

There is a range of current literature that explores various other clinical risk factors and, if applicable, their sensitivity and specificity as a predictor of IVH. A study showed factors such as small for gestational age, lower platelet crit levels (PCT), and lower Apgar scores at 5 minutes were all significantly associated with occurrences of IVH in comparison to non-IVH group infants. Upon further multivariate analysis, it was found that low PCT levels were the most predictive indicator of IVH. This was simply one variable in their larger model analyzing the role of platelet-related factors in morbidity and mortality of preterm infants toward common diseases; thus, sensitivity and specificity were not calculated [40]. In general, however, our results agreed with these findings in part as we were able to observe an association between lower Apgar scores at 5 minutes for IVH group infants prior to univariate analysis.

Additionally, studies regarding plasma activin A have also suggested the presence of lesser-utilized clinical indicators of IVH. With regards to the detection of early-stage (I and II) IVH in preterm infants, ​​a cutoff level of 0.8 μg/L activin A presented with a 100% specificity and sensitivity rate. This can point to neuroprotective measures being a beneficial intervention when concentration levels of plasma activin A in the infant's blood above that cutoff level are reached [41]. With respect to work done analyzing the role of Hct as a predictor, there are several studies detailing the high specificity and sensitivity of Hct as a predictor for other diseases such as ulcerative colitis [42], while others look into the sensitivities and specificities of mathematical models involving Hct as one of several variables [43]. As such, our work presents an opportunity to build upon Hct as an established predictor of disease which has shown use in being associated with a diagnosis of IVH prior as a group of variables.

Biological reasoning behind the association of Hct

The intricate relationship between Hct, blood flow, and viscosity is key to understanding the genesis of IVH, especially in ELBW infants. Hct is known to affect blood viscosity, which, in turn, influences CBF [44]. The immature germinal matrix, which is most abundant between 24 and 34 weeks of gestation, is susceptible to fluctuations in CBF. Our study identified that a day 2 Hct level of 35.42 is a significant predictor of IVH. A decrease in Hct, and thereby oxygen content, in the blood, necessitates an increase in CBF to maintain oxygen delivery [45]. This delicate equilibrium can be disturbed by changes in Hct levels, accelerating blood flow and increasing the risk of IVH, as suggested by Linder et al. and Mouqdad et al. [17,46]. Furthermore, this may lead to cerebral hypoperfusion, ischemia, and eventually, reperfusion-induced IVH [20]. Additionally, electrolyte levels in the blood, particularly serum sodium, can serve as another surrogate marker of blood flow and are implicated in the pathogenesis of IVH.

Prediction of IVH using multiple predictors

Anticipating IVH involves considering multiple predictors. Elevated PCO2 levels, indicative of hypercapnia, can predispose infants to IVH by causing vasodilation and a consequential increase in CBF [23-25]. Premature infants, lacking mature autoregulatory mechanisms, are particularly vulnerable to these fluctuations. Furthermore, outborn infants are found to be at a higher risk for IVH than their inborn counterparts. Antenatal steroid therapy, low 5-minute Apgar scores, and inadequate protein intake are also associated with heightened IVH risk. The administration of steroids to pregnant women serves to prevent RDS by inducing fetal lung maturity, potentially stabilizing CBF, and fortifying vascular structures in the germinal matrix [47,48]. However, surfactant treatment, while mitigating RDS, is another risk factor for IVH. Birth weight and serum sodium levels have also shown a significant correlation with IVH incidence. Hypernatremia-induced cerebral shrinkage, owing to increased osmolarity, can lead to vascular fragility and an elevated risk of IVH. Considering these variables in a multivariate model, accounting for potential confounding factors, can enhance the accuracy of IVH prediction [49,50].

Strengths and limitations

This case-control study offers novel insights into the association between Hct levels and severe IVH risk in extremely premature infants. The robust design, which matches cases and controls by gestational age, augments our understanding of IVH's potential mechanisms, highlighting Hct's potential as an early predictive biomarker. The rigorous use of both univariate and multivariate regression analyses also strengthens our findings.

However, inherent limitations of a retrospective case-control study, such as potential selection bias due to the single-center sample, restrict the generalizability of the findings. Additionally, the small sample size may limit statistical power and increase the chance of type II errors. Despite controlling for several confounding variables, unmeasured confounders may present residual confounding, necessitating these findings be confirmed in future prospective studies.

Future directions

To extend this research, larger multi-center studies are necessary for enhanced generalizability. These studies could elucidate further risk factors and underlying biological mechanisms. The integration of artificial intelligence (AI) could refine IVH prediction capabilities, using Hct and other variables to provide personalized risk scores. However, the application of AI tools necessitates rigorous validation and ethical considerations.

Moreover, investigating Hct-maintenance interventions could inform preventive strategies to reduce severe IVH incidence, thus improving clinical outcomes for extremely premature infants. Future research should, therefore, aim to explore, develop, and evaluate such interventions. While our study advances the understanding of severe IVH, further research is crucial to fully realize and leverage Hct's potential as a predictive marker.

## Conclusions

In conclusion, our study demonstrates the significance of decreasing Hct on the second day of life as a strong predictor for the occurrence of severe IVH (Grade III or IV) in extremely premature infants with a birth weight of less than 750 grams. The findings contribute to the growing body of knowledge surrounding the risk factors for IVH, potentially guiding advancements in prevention strategies and patient management. Future research should focus on validating these results in larger multicenter studies and developing advanced predictive models using machine learning to provide real-time, individualized risk assessments. Ultimately, such studies could lead to improved clinical outcomes for this vulnerable patient population.
